# Changes in Sources and Composition of Beach Waste in Coastal Cities around the Bohai Sea of China during the Tourist Peak and Off-Peak Seasons

**DOI:** 10.3390/ijerph20032573

**Published:** 2023-01-31

**Authors:** Tianqi Kong, Xuefei Li, Ke Pan, Wanli Zhang, Rundong Li

**Affiliations:** Key Laboratory of Clean Energy, School of Energy and Environment, Shenyang Aerospace University, No. 37 Daoyi South Avenue, Shenyang 110136, China

**Keywords:** beach waste, Bohai Sea, coastal city, source, composition

## Abstract

Beach waste is an important pollutant in the Bohai Sea and coastal cities around the Bohai Sea and has raised many social and environmental concerns in China. The semi-closed characteristics of the Bohai Sea, the well-developed tourism, the special industrial structure and residents’ living habits endow the beach waste around Bohai sea with a unique character that should be explored. This study investigated changes in the sources and composition of beach waste in coastal cities around the Bohai Sea of China during the tourist peak and off-peak seasons. Beach waste from twenty beaches in thirteen coastal cities around the Bohai Sea was sampled and analyzed in March and August 2021, respectively. The results showed that beach waste around the Bohai Sea was characterized by large quantities and small weights and was greatly affected by human coastal activities. The sources and composition of beach waste from different coasts and different seasons varied, whereas the overall trend was consistent. In terms of composition, beach waste in both the tourist peak season and off-peak season was mainly composed of plastics, fabrics and paper, which accounted for more than 70% of the total in weight. Meanwhile, the proportion of plastics in the total quantity of beach waste was greatest (maximum of up to 71%) and exhibited seasonal fluctuations, trending higher in the tourist peak season than in the off-peak season. In contrast, trends in the proportion of paper and fabrics in the total quantity and total weight of beach waste were relatively stable in different seasons. In terms of sources, beach waste mainly derived from human coastal activities, the proportion of which in the total quantity of beach waste in the tourist peak season reached 70.55% and was 11% higher than that in the tourist off-peak season. Shipping/fishing activities were the second largest source of beach waste, and their proportion in the total quantity of beach waste in the tourist peak season was 5% lower than that in the tourist off-peak season, as the tourist peak season around the Bohai Sea coincides exactly with the fishing moratorium. The quantity of smoking-related waste only accounted for 9.35% and 7.73% of beach waste in the tourist peak and off-peak seasons, respectively. The special semi-enclosed structure of the Bohai gulf, surrounded by land on three sides, aggravated the accumulation of beach waste on the coast. Source reduction and classified recovery, collaborative management of marine waste and beach waste, and joint prevention and control mechanisms of three provinces (Liaoning, Hebei and Shandong) and one municipality (Tianjin) were suggested for comprehensive management of beach waste in coastal cities around the Bohai Sea of China. This study provided valuable information for beach waste management in coastal cities around the Bohai Sea of China.

## 1. Introduction

The world’s oceans have been overexploited. Human activities are greatly affecting the marine and coastal environment and coastal ecosystems [1]. Large quantities of waste are dumped on the beaches or into the ocean. These forms of beach and marine waste can migrate over long distances, aided by wind, ocean currents, rivers, drainage and tsunami, and reach sea areas all over the world [2,3,4]. According to the United Nations Environment Program, beach waste poses a dire, vast and growing threat to the marine and coastal environments. It has been often reported that marine waste has negative effects on wildlife physiology, reproduction and diversity through ingestion and entanglement [5]. The waste piled on beaches greatly affects coastal sightseeing tourism and increases the cost of beach cleaning, especially in regions dominated by ocean tourism [6]. In addition, beach waste may bring harmful substances [7], pathogenic bacteria and invasive alien species [8]. However, current studies have mainly focused on marine waste such as marine micro plastics, seabed waste and floating waste [9,10], but beach waste has not attracted much attention [11]. Thus, a deep study concerning beach waste is necessary.

Beach waste is very complex and mainly composed of plastics, woods, metals, glass, rubber, clothing, foam and other materials [6,12]. Beaches, seabeds, and sea water everywhere are seriously endangered by plastic waste [13]. This plastic waste is left on beaches or washed onto beaches under the impacts of storms and waves. As a result, plastic waste accounts for a great proportion of beach waste. It was found that 60–80% of beach waste in some tourist cities in East China [14], Guangdong in South China [15], and the northern South China Sea [10] comprised plastics. With regard to sources of beach waste, the land and the sea are two main sources of beach waste, and the land-based sources are dominant [16,17]. Land-based sources of beach waste include beach recreation activities, inefficient solid beach waste management, and industry dumping into rivers that eventually reaches beaches [18,19]. Sea-based sources of beach waste mainly refer to marine activities, including shipping, ferries, fishing vessels, recreational boats and offshore installations [20], from which waste materials migrate onto the beaches [21].

Bohai Sea is the sole semi-closed marginal sea in China, with a sea area of about 78,000 km^2^, a mainland coastline of 2796 km, and an average water depth of 18 m. There are about forty rivers along its coast, including the Yellow River, Liaohe River and Haihe River. The Circum-Bohai-Sea region is an important strategic region for the coordinated development of Beijing, Tianjin and Hebei province in China, a significant ecological region for marine biodiversity protection as well as a vital functional region for the lives of local residents. Liaoning, Hebei, Shandong and Tianjin surround the Bohai sea, and their total gross marine output reached CNY 2621.9 billion, accounting for 31.4% of the national gross marine output of China. For decades, the Bohai Sea has paid a great price in its resources and environment to support the economic development around the Bohai region and has become the most degraded coastal area in China [22]. In particular, the semi-closed nature of the Bohai Sea results in a low water exchange ability with the outer sea and a poor self-purification capacity, which further aggravates the environmental pollution. Compared with other sea areas of China, the seawater quality of the Bohai Sea is the worst, and its average density of floating micro-plastics is the highest [23].

At present, most research studies concerning the environment around the Bohai Sea region have focused on its seawater quality [24], marine waste and their impacts on marine organisms [25,26]. Marine and beach waste are important pollutants affecting the Bohai Sea and its coastal cities and have raised many social and environmental concerns in China. However, there are no studies that have paid attention to beach waste in coastal cities around the Bohai Sea. However, the semi-closed characteristics of the Bohai Sea, the well-developed tourism, and especially the industrial structure and residents’ living habits have endowed the beach waste around the Bohai Sea with a unique character that should, therefore, be investigated. In this study, changes in the sources and composition of beach waste in coastal cities around the Bohai Sea of China during the tourist peak and off-peak seasons were investigated for the first time. Beach waste from twenty beaches in thirteen coastal cities around the Bohai Sea was sampled and analyzed in March and August 2021, respectively. This study provided valuable information for beach waste management in coastal cities around the Bohai Sea of China.

## 2. Materials and Methods

### 2.1. Study Area

**Figure 1:**
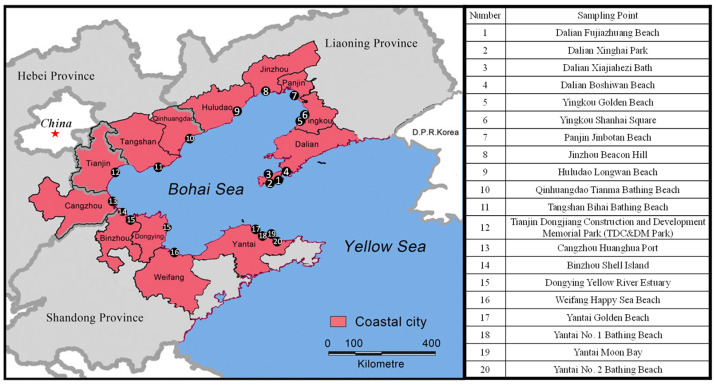
Study area and beach waste sampling points around the Bohai Sureea region.

**Figure 2:**
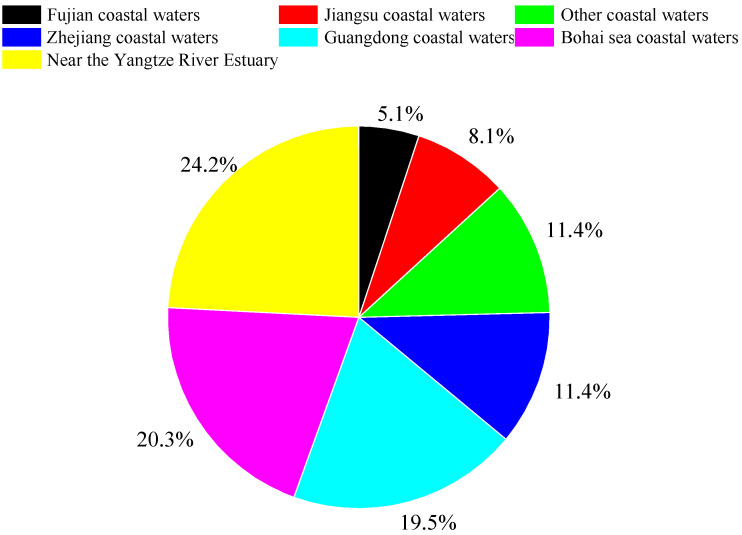
Distribution of marine dumping areas in China in 2021 [23].

The Bohai Sea is a semi-closed, shallow sea located deep inside the Chinese mainland (37°07′–41 north latitude, 117°35′–122°15′ east longitude), as shown in Figure 1. It is the largest inland sea of China, and its area is about 77,000 km^2^, accounting for 1.63% of the total territorial sea area of China. The Bohai Sea includes Liaodong Bay, Bohai Bay, Laizhou Bay, Central Sea Basin and Bohai Bay. It is surrounded by thirteen coastal cities (Dalian, Yingkou, Panjin, Jinzhou, Huludao, Qinhuangdao, Tangshan, Cangzhou, Tianjin, Binzhou, Dongying, Weifang, Yantai) belonging to Liaoning, Hebei, Tianjin, and Shandong provinces. The Bohai Sea has a unique natural ecology, obvious geographical advantages and a prominent strategic position. Meanwhile, it is confronted with many challenges, including unstable environmental quality, a degraded ecosystem and high environmental risks. The Bohai Sea is a major ocean dumping area, accounting for 20.3% of the total amount of marine waste dumped in China, as shown in Figure 2. The beaches of these thirteen coastal cities around the Bohai Sea were selected as the study area, which covered different coastal types, including bedrock coast, sandy coast, silty coast, and muddy coast.

### 2.2. Experimental Procedures

The sampling points are shown in Figure 1. Beach waste from twenty beaches in thirteen coastal cities around the Bohai Sea was sampled and analyzed in the tourist off-peak season (1 March to 15 March) and the tourist peak season (1 August to 15 August) in 2021, respectively. The beach waste samples were sealed and stored in plastic bags.

### 2.3. Analytical Methods

After collection, samples of beach waste from each sampling point during the tourist peak and off-peak seasons were sorted, counted and weighed, respectively. The composition of these beach waste samples was divided into nine categories: plastic, wood products, paper, metal, polystyrene plastic foam, rubber, fabric, glass, and others. According to the Northwest Pacific Organization, the sources of beach waste were divided into five categories (Table 1): human coastal activities, shipping/fishing activities, medical/personal hygiene, smoking-related activities, and other discards.

## 3. Results and Discussion

### 3.1. Composition of Beach Waste

**Figure 3:**
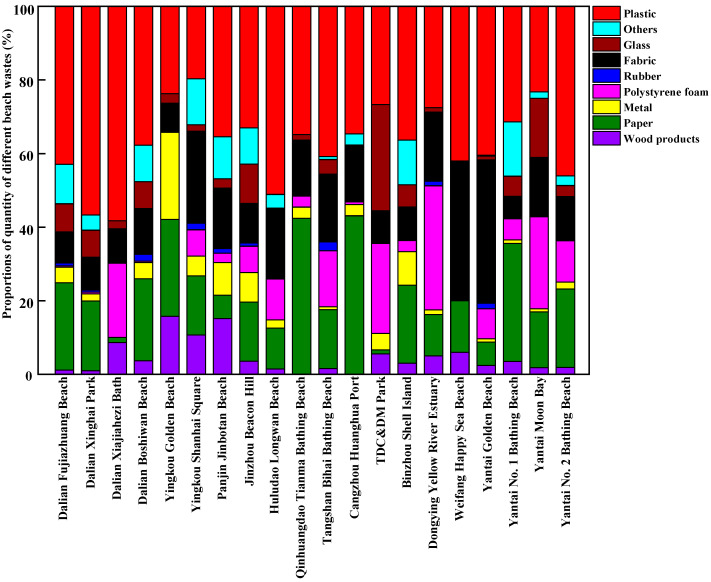
The proportions of different components of beach waste in coastal cities around the Bohai Sea of China in the tourist off-peak season, by quantity.

**Figure 4:**
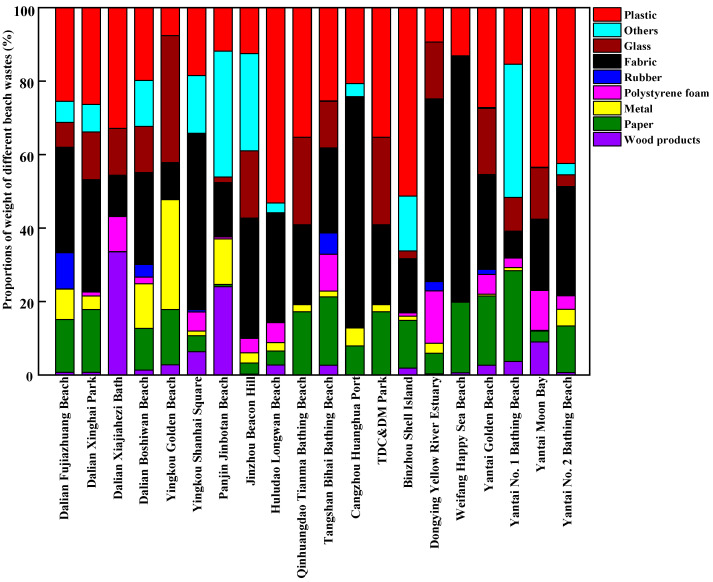
The proportions of different components of beach waste in coastal cities around the Bohai Sea of China in the tourist off-peak season, by weight.

**Figure 5:**
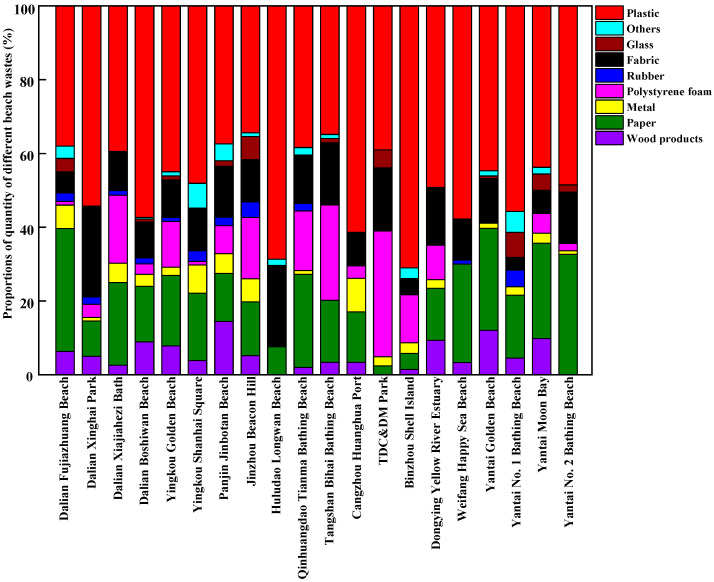
The proportions of different components of beach waste in coastal cities around the Bohai Sea of China in the tourist peak season, by quantity.

**Figure 6:**
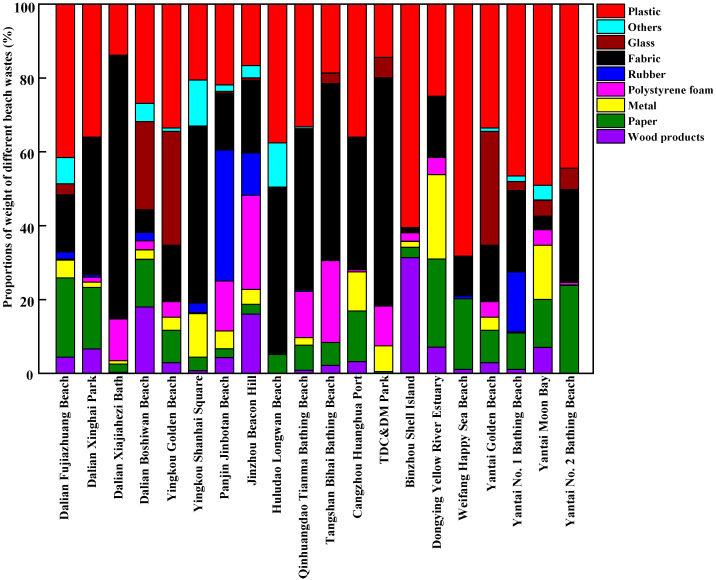
The proportions of different components of beach waste in coastal cities around the Bohai Sea of China in the tourist peak season, by weight.

The composition of beach waste obtained from twenty beaches in thirteen coastal cities around the Bohai Sea of China during the tourist off-peak and peak seasons is shown in Figure 3, Figure 4, Figure 5 and Figure 6, respectively. The proportions by quantity and weight of each type of beach waste were measured separately. The results showed that plastic waste appeared as one of the major components of beach waste around the Bohai Sea whether in the tourist off-peak or peak season. The average proportions by quantity and weight of plastic materials in beach waste from twenty beaches in the tourist off-peak season reached 27.11% and 26.34%, respectively, whereas the corresponding values in the tourist peak season further increased to 48.34% and 34.68%, respectively. This plastic beach waste mainly included plastic packaging bags, plastic bottles, bottle caps and other plastic products, which indicated that the composition of beach waste was governed by human activities. Plastics are often reported as the most common types of beach waste all over the world [27]. Except for the impact of human activities, the properties of plastics and environmental factors also played an important role in the global migration of plastics [28]. Due to the low density and poor degradability of plastics, they can float in the ocean for a long time and might be transported over great distances by waves and currents [29]. Meanwhile, the semi-closed nature of the Bohai Sea further aggravated the accumulation of plastics in its seawater. In the tourist off-peak season, tourist numbers greatly decreased. The key factors governing plastic beach waste were migratory flows induced by winds and storms. As a result, the average quantity and weight proportions of plastic beach waste in the tourist off-peak season declined by 21.23% and 8.34%, respectively, compared with their levels in the tourist peak season.

Following plastics, paper waste and fabrics were also major components of beach waste around the Bohai Sea. In terms of average quantity, the proportion of waste paper in beach waste from twenty beaches in the tourist off-peak and peak season reached 18.32% and 17.97%, respectively. These waste paper components in beach waste mainly included food packaging, towels and cigarette boxes discarded by tourists. Meanwhile, in terms of average weight, the proportion of fabrics in the tourist off-peak and peak season remained relatively stable, reaching 28.65% and 27.28%, respectively. Polystyrene foam is a key material in fishing net buoys and life vests and is often found in beach waste. The percentages of polystyrene foam in beach waste from twenty beaches in thirteen coastal cities were highly variable and random. Among them, the percentages of polystyrene foam by weight and quantity were highest in Dongjiang Construction and Development Memorial Park of Tianjin, reaching 21.40% and 24.44% in the tourist off-peak season and 10.81% and 34.15% in the tourist peak season, respectively. The perniciousness of polystyrene foams to the marine environment and marine organisms is not less than that of plastic waste. The polystyrene foams are broken up and disintegrated into many pieces of micro-plastic by mechanical (wind and wave), biological (microorganisms) and chemical (ultraviolet radiation) degradation [30]. Waste wood products, metals, and rubber materials only accounted for small proportions of the beach waste from most sampling points. Waste wood products were mainly produced from fishery activity and maritime transport, and their proportions in beach waste in the tourist off-peak and peak seasons exhibited no significant differences. Waste rubber materials were not often found in beach waste, but Jinbotan Beach of Panjin was an exception. By weight, the proportion of rubber materials in beach waste on Jinbotan Beach in the tourist peak season reached 35.46%. Metals in beach waste mainly comprised cans, some metal fragments related to maritime transport, and metal toys discarded by tourists.

### 3.2. Sources of Beach Waste

**Figure 7:**
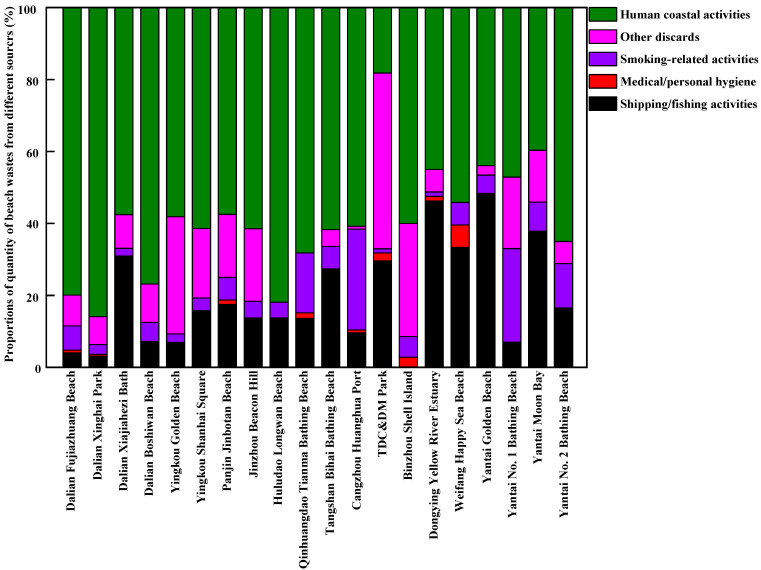
The proportions of beach waste from different sources in coastal cities around the Bohai Sea of China in the tourist off-peak season, by quantity.

**Figure 8:**
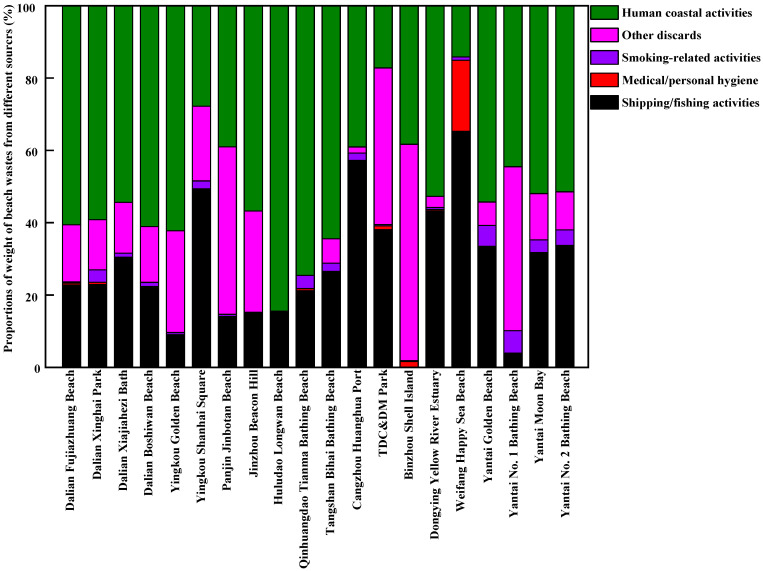
The proportions of beach waste from different sources in coastal cities around the Bohai Sea of China in the tourist off-peak season, by weight.

**Figure 9:**
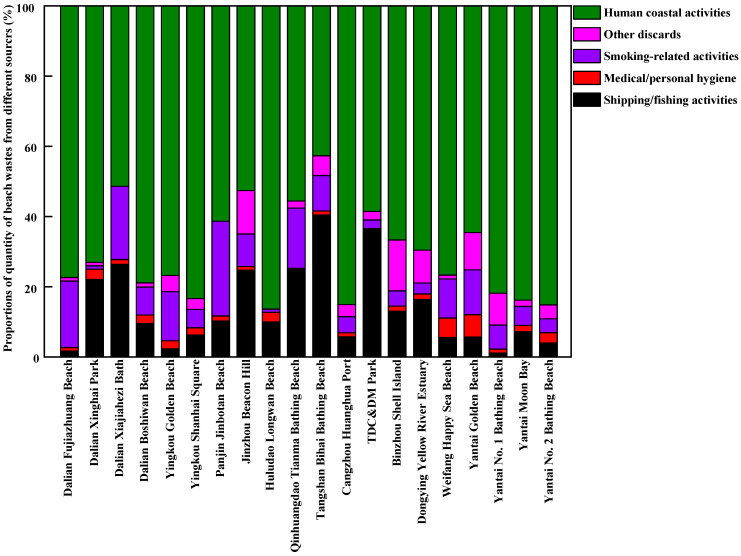
The proportions of beach waste from different sources in coastal cities around the Bohai Sea of China in the tourist peak season, by quantity.

**Figure 10:**
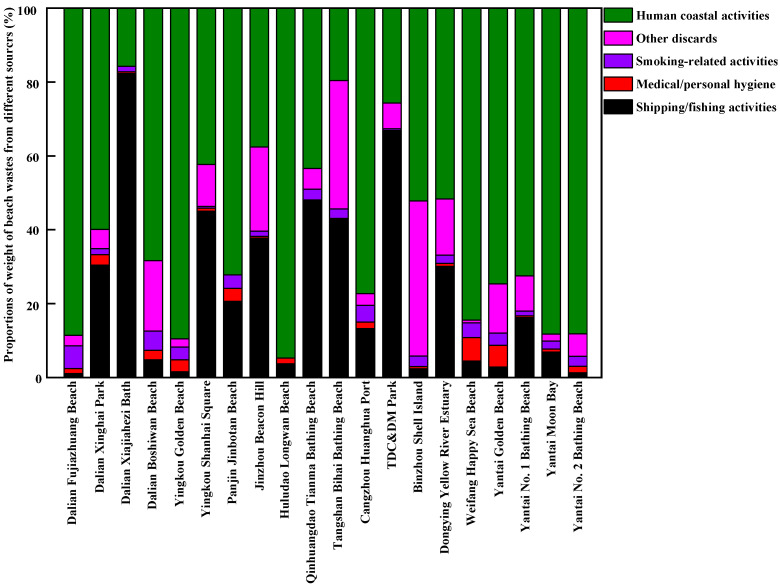
The proportions of beach waste from different sources in coastal cities around the Bohai Sea of China in the tourist peak season, by weight.

The sources of beach waste in samples obtained from twenty beaches in thirteen coastal cities around the Bohai Sea of China during the tourist off-peak and peak seasons are presented in Figure 7, Figure 8, Figure 9 and Figure 10, respectively. The proportions, by quantity and weight, from each source of beach waste were assessed separately. It was found that overall, human coastal activity was the greatest source of waste on most beaches, which mainly included food packaging bags, clothing, beach toys, tissues, metal cans, plastic and glass bottles, etc. In terms of average quantity and weight, the proportions of beach waste from human coastal activity at twenty beaches in the tourist off-peak season reached 59.22% and 50.40%, respectively. In the tourist peak season, the corresponding values were higher, reaching 70.55% and 62.35%, respectively. This was in agreement with the finding that human coastal activities were the dominant source of beach waste in other coastal areas around the world [17,31]. In particular, on a weight basis, the percentage of human coastal activity-based beach waste on Dalian Fujiazhuang Beach, Yingkou Golden Beach, Huludao Longwan Beach, Yantai Moon Bay and No. 2 Bathing Beach in the tourist peak season was close to or exceeded 90%. This result was in line with the finding that the densities of beach waste are greatly increased by up to 40% in the summer with the increase in the number of tourists [32]. In the tourist peak season, these coastal tourist cities around the Bohai Sea are under pressure from tourists, which results in higher proportions of beach waste from human coastal activity at most sampling beaches. However, Dalian Xiajiahezi Bath, Huludao Longwan Beach, Qinhuangdao Tianma Bathing Beach and Tangshan Bihai Bathing Beach were the exceptions. At these beaches, the proportions of beach waste from human coastal activity in the tourist off-peak season were not lower than those in the tourist peak season. The reason might be that the semi-closed nature of the Bohai Sea led to lower water exchange ability with the outer sea. As a result, the amount of marine waste might accumulate in seawater for long periods and be washed up onto beaches. Land-based activities are the main source of beach waste. It was reported that 80% of beach waste came from the land, and 20% from the sea [27]. Fishing gear waste was the main sea-based form of waste, and it very dangerous because it might entangle corals, turtles and other marine organisms [31].

The second greatest source of beach waste was shipping/fishing activity. This mainly included buoys, ropes and fishing nets. By quantity and weight, the average proportions of beach waste from shipping/fishing activity on these twenty beaches during the tourist off-peak season reached 19.12% and 27.75%, respectively, which were higher than their levels during the tourist peak season (13.71% in quantity and 23.10% in weight, respectively). One reason was that the tourist peak season between July to September for coastal cities around Bohai Sea coincided exactly with the seasonal fishing moratorium on the Bohai Sea. At that time, shipping/fishing activities were greatly decreased, which reduced their contribution as a source of beach waste. Meanwhile, the frequency of beach pollution cleanup operations was much higher during the tourist peak season than during the tourist off-peak season. These buoys, ropes and fishing nets washed ashore by the seawater would not stay on the beach for very long before they were cleaned up during the tourist peak season. Moreover, during the lengthy tourist off-peak season, a great deal of marine waste (including many buoys and fishing nets) would be washed ashore by strong winds, waves and ocean currents.

Counting as the third greatest source of beach waste were other discarded materials, including construction waste and other debris from unidentifiable sources. In terms of average quantity and weight, the proportions of other discarded materials in beach waste during the tourist off-peak season on these twenty sampled beaches were also higher than their levels during the tourist peak season. Many waste materials were degraded into unidentified debris by waves. In particular, by weight, the proportions of other discarded materials in total beach waste at Panjin Jinbotan Beach, TDC&DM Park, Binzhou Shell Island and Yantai No. 1 Bathing Beach even reached above 40% during the tourist off-peak season. From Figure 7, Figure 8, Figure 9 and Figure 10, the fourth greatest source of beach waste was tobacco smoking-related activity. These lighters, cigarettes, cigarette butts and filters often appeared as the most common beach waste on beaches all over the world [28,33]. In the tourist off-peak season of Cangzhou Huanghua Port, the proportion of smoking-related materials in beach waste was the highest, at up to 28% in terms of quantity. In general, the proportions of medical/personal hygiene-related forms of beach waste (such as plastic syringes, diapers, cotton swabs, sanitary pads, toothbrushes) at most sampled beaches were relatively low. During the tourist off-peak season, medical/personal hygiene-related beach waste materials were not found at more than half of the sampling points. During the tourist peak season, disposable surgical masks appeared as the dominant medical/personal hygiene-related beach waste due to the prevalence of COVID-19.

### 3.3. Current Status of Beach Waste Management throughout the Bohai Sea Region

Currently, marine and beach waste are important pollutants for the Bohai Sea and its surrounding coastal cities and have spurred many social and environmental concerns in China. Due to the semi-closed nature of the Bohai Sea and its low water exchange ability with the outer sea, marine waste and beach waste are exchanged only within a certain range of time and space. Thus, marine and beach waste should be taken into account together. According to the 2021 China Marine Ecological Environment Status Bulletin [23], the average quantity of beach waste in 51 coastal regions in China reached 154,816 pieces/km^2^, and the average density was 1849 kg/km^2^. It was reported that plastic waste (75.9%) was the overwhelming type of beach waste. China has been highly attentive the issue of marine waste pollution and has already issued a series of relevant laws, regulations and documents concerning marine environmental protection and marine waste management, as shown in Table 2 and Table 3. The semi-closed nature of the Bohai Sea endows it with a special ecological environment different from that of other sea areas. Meanwhile, due to its prominent strategic position, the Bohai Sea has been selected as a demonstration plot for marine environmental governance. In 2018, the National Development and Reform Commission of China issued Action Plan for the Comprehensive Governance of the Bohai Sea, with clearly stated relevant measures for the prevention and control of marine pollution, including waste in rivers entering the Bohai Sea and coastal waters. As an important source of marine waste, beach waste deserves to be regulated normatively [34,35].

**Figure 11:**
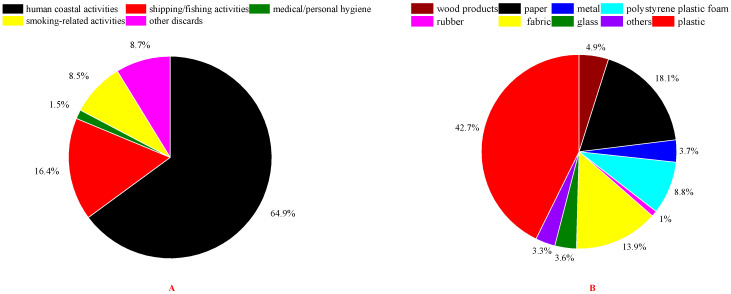
The average proportions of different components and sources of beach waste of twenty beaches in thirteen coastal cities around the Bohai Sea of China: sources of beach waste (**A**); components of beach waste (**B**).

The quantitative proportions of different components and sources of beach waste on twenty beaches during the tourist off-peak and peak seasons were averaged to approximate the beach waste situation for the whole year in these thirteen coastal cities around the Bohai Sea of China, as shown in Figure 11. It was found that plastic waste accounted for 42.7% of the total quantity of beach waste around the Bohai Sea, which was much lower than the national averages reported in 2019 (75.9%), 2020 (84.6%) and 2021 (81.7%) for China in the China Marine Ecological Environment Bulletin. Investigations concerning plastic beach waste on Shilaoren Beach in Qingdao [12] and around the northern South China Sea [11] obtained similar results (56.7% and 42.0%, respectively). These results might be related to the low accumulation rate of plastic beach waste around the Bohai Sea. It was reported that the accumulation rate of plastic beach waste around the Bohai Sea was only 0.12 pieces/m^2^/cycle; that was the lowest in four major sea areas (Yellow Sea, Bohai Sea, East Sea and South Sea) of China [23]. In recent years, various countries and regions have successively promulgated “plastic restriction orders” and “plastic ban orders” concerning plastic products. According to Opinions on Further Strengthening the Treatment of Plastic Pollution issued by China in 2020, the production and sales of non-degradable plastic bags, disposable plastic tableware, disposable plastic products in hotels, express plastic packaging and polyethylene agricultural mulch films were restricted, which further expanded the scope of the plastic restriction order promulgated before 2019. However, the large quantity of plastics in beach waste still reflected the seriousness of the current plastic waste problem. Meanwhile, paper waste accounted for 18.1% of the quantity of beach waste around Bohai Sea, which was much higher than the national average reported in 2020 (4.1%) and 2021 (11.3%) by the China Marine Ecological Environment Bulletin. It was followed by waste fabrics (13.9%) and polystyrene foam (8.8%). Meanwhile, five components (waste wood, metals, glass, rubber and other materials) each accounted for percentages of less than 5% on a quantity basis. Among them, the quantity of wood products (4.9%) in beach waste around Bohai Sea was close to the national average for China in 2020 (4.1%) and 2021 (5.7%).

**Figure 12:**
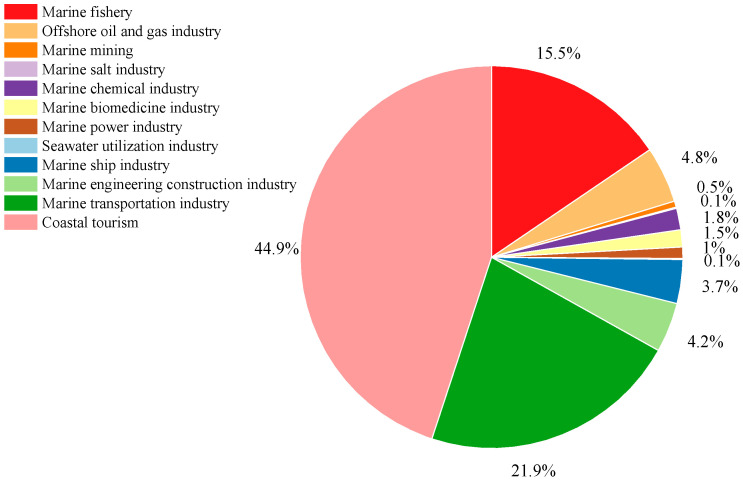
Contributions by major marine industries to the GDP of China’s marine economy in 2021 [36].

As shown in Figure 11A, beach waste in coastal cities around the Bohai Sea mainly derived from human coastal activities (64.9%), which might be closely related to the rapid development of coastal tourism. According to the China Marine Economic Statistical Bulletin [36], coastal tourism was the predominant marine economy in China. As shown in Figure 12, in 2021 coastal tourism accounted for 44.92% of the total gross domestic product (GDP) of the marine economy in China. The shipping and fishing industries together contributed 19.27% of the marine economy’s GDP and were also among the major marine industries in China. This result was in line with the finding that shipping/fishing activities are the second largest source of beach waste after human coastal activities. The fast development of coastal tourism and the shipping and fishing industries led to the aggravation of beach waste pollution and also raised a series of environmental concerns that should be urgently addressed.

The waste scattered on beaches has a major negative impact on the natural landscape and the ecological environment of the coastal region. The high mobility of beach and marine waste materials due to frequent winds, waves, ocean currents and tsunami has further magnified their perniciousness.

In particular, the Bohai Sea is the sole semi-closed marginal sea in China. There are about forty rivers along its coast, including the Yellow River, Liaohe River and Haihe River. The Bohai Sea is surrounded by Liaoning, Hebei, Shandong and Tianjin and is a vital functional region for the lives of local residents as well as a significant ecological region for marine biodiversity protection. The semi-closed nature of the Bohai Sea, the well-developed tourism, the special industrial structure and residents’ living habits endow the beach waste around Bohai Sea with a unique character. In order to control the beach and marine waste pollution of coastal cities around the Bohai Sea, a series of suggestions are proposed as follows:

(1) Source reduction and classified recovery. The production, sale and use of disposable plastic products should be further restricted. Reusable tools and products should be encouraged in shipping and fishing activities. The behaviors of individuals (tourists and marine industry practitioners) and relevant enterprises must be well-regulated. The random waste dumping into the sea and on beaches must be strictly prohibited. The identification of the sources, components and distribution of beach waste around the Bohai Sea should be well-investigated. The classified recovery of beach waste is indispensable. Valuable components and materials in beach waste should be recycled.

(2) Collaborative management of marine waste and beach waste. Marine waste and beach waste will exchange positions within a certain range of time and space. Beach waste management should also be incorporated into the comprehensive governance of the Bohai Sea. Laws and regulations concerning collaborative management of marine waste and beach waste should be established. Deeper insights into the whole life cycle of beach waste combined with marine waste should be developed.

(3) Joint prevention and control mechanisms in three provinces (Liaoning, Hebei and Shandong) and one municipality (Tianjin). The Bohai Sea is surrounded by Liaoning, Hebei, Shandong and Tianjin. These four regions must keep pace in the comprehensive governance of marine waste and beach waste in the Bohai Sea. The joint prevention and control mechanisms of the three provinces and one municipality for marine waste and beach waste pollution are indispensable. With the efforts of the government, enterprises, institutions, environmental protection organizations and the public, the three provinces and one municipality will form a leading force for beach waste prevention and control.

## 4. Conclusions

Beach waste around the Bohai Sea was characterized by large quantities of small, lightweight materials and was greatly affected by human coastal activities. The sources and components of beach waste from different coasts and in different seasons were varied, whereas the overall trend was consistent. In terms of composition, beach waste in both the tourist peak season and off-peak season mainly of plastics, fabrics and paper, which together accounted for more than 70% of the total in weight. Meanwhile, the proportion of plastics in the total quantity of beach waste was the greatest (up to a maximum of 71%) and exhibited seasonal fluctuations that were higher in the tourist peak season than in the tourist off-peak season. In contrast, the percentages of paper and fabrics in the total quantity and total weight of beach waste maintained relatively stable trends across different seasons. In terms of sources, beach waste was mainly derived from human coastal activities, which accounted for 70.55% of the total quantity of beach waste during the tourist peak season, 11% higher than that in the tourist off-peak season. Shipping/fishing activities were the second largest source of beach waste, and their share of the total quantity of beach waste during the tourist peak season was 5% lower than that during the tourist off-peak season, as the tourist peak season around the Bohai Sea coincides closely with the seasonal fishing moratorium. Smoking-related waste only accounted for 9.35% and 7.73% of the quantity of beach waste during the tourist peak and off-peak seasons, respectively. The special semi-enclosed structure of the Bohai gulf surrounded by land on three sides aggravated the accumulation of beach waste on the coast. Source reduction and classified recovery, collaborative management of marine waste and beach waste, and joint prevention and control mechanisms of the three provinces (Liaoning, Hebei and Shandong) and one municipality (Tianjin) were suggested for comprehensive governance of beach waste in coastal cities around the Bohai Sea. Although this study provided valuable information concerning sources and components of beach waste in coastal cities around the Bohai Sea, the physical, chemical and biochemical properties of beach waste are still not clear. In the future, the characteristics of beach waste should be further investigated, and the treatment technologies should be taken into consideration.

## Figures and Tables

**Table 1:** Classification of beach waste sources.

Source	Composition
Human coastal activities	Plastic bottles, plastic bags, toys, plastic tableware, food packaging containers, clothes, shoes, metal drink cans and related materials, paper beach waste, glass bottles, etc.
Shipping/fishing activities	Buoys, beacons, fishing gear, etc.
Medical/personal hygiene	Plastic syringes, diapers, cotton swabs, sanitary pads, toothbrushes
Smoking-related activities	Lighters, cigarettes, cigarette butts, filters
Other discards	Home appliances and electronic products, batteries, processed wood, rubber tires, metal cans, barrels, gas cylinders (>4 L), glass bulbs, lampshades and tubes, glass and ceramic building materials, cloth carpets and cloth furniture, etc.

**Table 2:** Current laws and regulations concerning marine and beach waste management in China.

Laws and Regulations	Implementation/Promulgation Date	Last Revision Date
Marine Environmental Protection Law of the People’s Republic of China	1983	2017
Regulations of the People’s Republic of China on Environmental Protection in Offshore Oil Exploration and Development	1983	-
Regulations of the People’s Republic of China on the Administration of Marine Dumping	1985	-
Regulations of the People’s Republic of China on Prevention of Environmental Pollution Caused by Shipbreaking	1988	2016
Regulations of the People’s Republic of China on the Prevention and Control of Pollution Damage to Marine Environment by Land-based Pollutants	1990	-
Law of the People’s Republic of China on Prevention and Control of Beach Waste Pollution in Hometown	1996	2020
Regulations of the People’s Republic of China on Prevention and Control of Pollution Damage to Marine Environment by Marine Engineering Construction Projects	2006	-
Island Protection Law of the People’s Republic of China	2010	-
Regulations of the People’s Republic of China on the Prevention and Control of Marine Environment Pollution by Ships	2010	2017
Notice on Accelerating the Classification of Domestic Beach Waste in Some Key Cities	2017	-
Opinions on Further Strengthening Plastic Pollution Control	2020	-

**Table 3:** Environmental policies and plans related to oceans in China.

Environmental Policies and Plans	Publishing Authority	Promulgation Time
The 14th Five-Year Plan for Marine Ecological Environmental Protection	Ministry of Ecology and Environment, Development and Reform Commission, Ministry of Natural Resources, Ministry of Transport, Ministry of Agriculture and Rural Affairs, China Coast Guard	2020
Action Plan for Comprehensive Governance of Key Sea Areas	Ministry of Ecology and Environment, Development and Reform Commission, Ministry of Natural Resources, Ministry of Housing and Urban-Rural Development, Ministry of Transport, Ministry of Agriculture and Rural Affairs, China Coast Guard	2022
Regional Ecological Quality Evaluation Measures	Ministry of Ecology and Environment	2021
Technical Guidelines for Marine Ecological Restoration (Trial)	Ministry of Natural Resources	2021
Opinions on Strengthening the Supervision of Marine Aquaculture Ecological Environment	Ministry of Ecology and Environment, Ministry of Agriculture and Rural Affairs	2022
Opinions on Comprehensively Strengthening Ecological Environmental Protection and Resolutely Fighting the Tough Battle of Pollution Prevention and Control	State Council	2018
Water Pollution Prevention and Control Plan for Key River Basins (2016–2020)	Ministry of Environmental Protection, National Development and Reform Commission, Ministry of Water Resources	2017
Pollution Prevention and Control Plan for Nearshore Sea Areas	Ministry of Environmental Protection, Development and Reform Commission, Ministry of Science and Technology, Ministry of Industry and Information Technology, Ministry of Finance, Ministry of Housing and Urban-Rural Development, Forestry Bureau, Oceanic Bureau	2017
Thirteenth Five-Year Plan for Ecological Environmental Protection	State Council	2016
Water Pollution Prevention and Control Action Plan	State Council	2015
Action Plan for the Comprehensive Governance of Bohai Sea	National Development and Reform Commission	2018

## Data Availability

The data presented in this study are available on request from the corresponding author. The data are not publicly available due to privacy.
